# Combining convolutional neural networks and self-attention for fundus diseases identification

**DOI:** 10.1038/s41598-022-27358-6

**Published:** 2023-01-02

**Authors:** Keya Wang, Chuanyun Xu, Gang Li, Yang Zhang, Yu Zheng, Chengjie Sun

**Affiliations:** 1 School of Artificial Intelligence, Chongqing University of Technology, Chongqing, 401135 China; 2 College of Computer and Information Science, Chongqing Normal University, Chongqing, 401331 China

**Keywords:** Computational biology and bioinformatics, Computational neuroscience, Image processing, Machine learning

## Abstract

Early detection of lesions is of great significance for treating fundus diseases. Fundus photography is an effective and convenient screening technique by which common fundus diseases can be detected. In this study, we use color fundus images to distinguish among multiple fundus diseases. Existing research on fundus disease classification has achieved some success through deep learning techniques, but there is still much room for improvement in model evaluation metrics using only deep convolutional neural network (CNN) architectures with limited global modeling ability; the simultaneous diagnosis of multiple fundus diseases still faces great challenges. Therefore, given that the self-attention (SA) model with a global receptive field may have robust global-level feature modeling ability, we propose a multistage fundus image classification model MBSaNet which combines CNN and SA mechanism. The convolution block extracts the local information of the fundus image, and the SA module further captures the complex relationships between different spatial positions, thereby directly detecting one or more fundus diseases in retinal fundus image. In the initial stage of feature extraction, we propose a multiscale feature fusion stem, which uses convolutional kernels of different scales to extract low-level features of the input image and fuse them to improve recognition accuracy. The training and testing were performed based on the ODIR-5k dataset. The experimental results show that MBSaNet achieves state-of-the-art performance with fewer parameters. The wide range of diseases and different fundus image collection conditions confirmed the applicability of MBSaNet.

## Introduction

Fundus disease can cause vision loss and, as the disease progresses, blindness. Currently, common fundus diseases that affect visual function include diabetic retinopathy (DR), age-related macular degeneration (AMD), and glaucoma. The progression of fundus diseases to advanced stages often severely affects the visual function of patients, and there is no specific treatment for such diseases. A significant portion of the world’s population suffers from diabetes. DR is the most common complication of diabetes, with no obvious abnormal symptoms in the early stages but can eventually cause blindness. DR is one of the four major blindness diseases in^1^. If DR is detected at an early stage, patients usually receive a good prognosis for treatment. Moreover, glaucoma is an irreversible neurodegenerative eye disease and is considered a leading cause of visual disability in the world^2^. According to the World Health Organization, there will be up to 78 million glaucoma patients globally by 2020^3^. Therefore, early detection and treatment of fundus diseases are crucial. Artificial intelligence technology can help ophthalmologists make accurate diagnoses based on comprehensive medical data and provide new strategies to improve the diagnoses and treatments of eye diseases in primary hospitals.

Recently, some proposed convolutional neural network (CNN) -based models have achieved state-of-the-art (SOTA) performance in tasks, such as image classification and object detection, e.g., VGGNet^4^, ResNet^5^, GoogLeNet^6^, and EfficientNet^7^. Some have also been applied to fundus disease identification. Meanwhile, with the success of self-attention (SA) models such as Transformer^8^ in natural language processing^9,10^, several scholars have attempted to introduce SA mechanisms into computer vision (CV). Recently, Vision Transformer (ViT)^11^ has shown that almost only a single vanilla Transformer layer is required to achieve decent performance on ImageNet-1K^12^. Particularly, ViT achieved comparable results to SOTA CNNs when pretrained on the large-scale private JFT-300M dataset^13^, indicating that the Transformer model has higher model capacity than CNNs. Moreover, although Transformer architectures are becoming increasingly well-known in vision tasks and have shown quite competitive performance compared with CNN architectures in various vision tasks^14,15^, the excellent performance is implemented on the premise of having considerable training data support; Transformer architectures still lag behind CNNs under low data volume conditions. Therefore, Transformer-based models have not yet been applied to the field of fundus disease classification with a small sample size.

At present, the research on fundus image classification still has the following challenges. First, the classification of multilabel fundus images is a common practical problem, because a real fundus image is likely to contain multiple fundus diseases. Second, under the conditions of limited fundus image data and unavoidable image noise, it is difficult to obtain a model with high disease detection accuracy using a pure deep CNN architecture. Therefore, for the first problem, we use a problem transformation-based approach that transforms the multilabel classification problem for each image into a two-class classification problem for each label. For the second problem, owing to the poor performance of a single CNN model, the current optimal solutions almost all use the method of integrating multiple CNN models, such as^16,17^. Although a better and more comprehensive robust classifier can be obtained by integrating multiple weak classifiers, the inference cost will increase significantly. In this regard, we adopt the most remarkable strategy of this study: integrating the CNN (particularly the MBConv block) and the Transformer into the same network architecture.

Since the convolutional layer has a strong inductive bias prior and has a better convergence speed, and the self-attention layer with a global receptive field has a stronger feature modeling ability, which can compensate for the lack of global modeling capability of the convolutional layer. Therefore, they are considered to be integrated into the same multistage feature extraction backbone network. Convolution is used to extract low-level local features, and the Transformer captures long-term dependencies. By combining CNN architecture with stronger generalization performance and Transformer architecture with higher model capacity and stronger learning ability, the model can achieve better generalization performance and stronger learning ability, making it more suitable for fundus image classification tasks. In addition, because such a model is usually deployed on mobile devices, considering the computational efficiency, we only turn on the global receptive field when the size of the feature map reaches a manageable level after downsampling, which is similar to the real situation.

Networks containing Transformer architectures perform poorly on undersized datasets due to their lack of the inductive bias that CNN architectures have^18^. In our experiments, we applied data augmentation to our dataset, mainly to alleviate the overfitting phenomenon of the network, and by transforming training images, we can obtain a network with better generalisation capability. Inspired by CoAtNet^18^, we propose a multistage feature extraction backbone network -MBSaNet -which combines convolutional blocks and SA modules, for identifying multiple fundus diseases.

## Related Work

### Fundus disease identification method

Fundus photography is a common method for fundus disease examination; Compared with other examination methods such as fundus fluorescein angiography (FFA) and fundus Optical Coherence Tomography (OCT), it has the advantages of low cost, fast detection speed, and simple image acquisition. In recent years, with the continuous advancement of CV and image processing technology, disease screening and identification methods based on fundus images have emerged. Considering the characteristics of image datasets, a shallow CNN^19^ was designed for automatic detection of age-related macular degeneration (AMD), the average accuracy of ten-fold cross validation was 95.45$\%$, and the average accuracy of blindfold was 91.17$\%$.^20^ employed the Inception-v3 structure to diagnose diabetic retinopathy, trained on 128,175 fundus images, and then demonstrated good results on two validation datasets, demonstrating that deep learning technology can be applied to ophthalmic illness diagnoses. Based on EfficientNet, a model integration strategy was proposed^16^, inputting the color and gray versions of the same fundus image into two EfficientNets with the same architecture for training, and finally integrating the output results of the two models to obtain the final output. Considering the possible correlation between the fundus images of both eyes of the same patient, a dense correlation network (DCNet)^21^ was devised to aggregate related characteristics based on the dense spatial correlation between paired fundus pictures. Several alternative backbone feature extraction networks are employed for trials on the ODIR-5K dataset, indicating that the fusion has been completed. The DCNet module effectively improved the recognition accuracy of fundus illnesses, according to the trial data. To extract the depth features of the fundus images,^22^ used the R-CNN+LSTM architecture. The classification accuracy was enhanced by 4.28$\%$ and 1.61$\%$, respectively, by using the residual method and adding the LSTM model to the RCNN+LSTM model. In terms of feature selection, the 350 deep features are subjected to a multi-level feature selection approach known as NCAR, which improved accuracy and reduced the support vector machine (SVM) classifier’s computation time. For the detection of glaucoma, diabetic retinopathy and cataracts from fundus images, three pipelines^23^ were built in which twelve deep learning models and eight support vector machine (SVM) classifiers were trained, using different pretrained models such as Inception-v3, Alexnet, VGGNet and ResNet. The experimental results show that the inception-v3 model had the best performance with an accuracy of 99.30$\%$ and an f1-score of 99.39$\%$.^24^ employed transfer learning to classify diabetic retinopathy fundus images. Experiments on the DR1 and MESSIDOR public datasets indicated that knowledge learned in other large datasets (source domain) could be better classified in small datasets (target domain) via transfer learning.^25^ developed an enhanced residual dense block CNN, which could effectively classify fundus images into “good quality” and “low quality” to avoid delaying patient treatment and solve the problem of quality classification of fundus images.^26^ offered a six-level cataract grading method that focuses on multifeature fusion and extracted features from the residual network (ResNet-18) and gray-level cooccurrence matrix (GLCM), with promising results.

### Transformer architecture

Transformer^8^ is an attention-based encoder-decoder architecture that has revolutionized the field of natural language processing. Recently, inspired by this major achievement, several pioneering studies have been carried out in the computer vision (CV) field, demonstrating their effectiveness in various CV tasks. With competitive modeling capabilities, VITs achieve impressive results on multiple benchmarks such as ImageNet, COCO, and ADE20k, compared with existing CNNs. As the spearheading work of Transformer within the CV field, the visual Transformer (ViT)^11^ structure can accomplish fabulous performance on ImageNet. Be that as it may, an impediment of ViT is the requirement for large-scale datasets, such as ImageNet-21k^12^ and JFT-300M^12^ (which may be a private dataset), to obtain pretrained models. In spite of the fact that SA modules are able to improve recognition accuracy, they more often than not bring about extra computation and are hence frequently seen as add-ons to CNNs, similar to the squeeze-and-excitation (SE)^27^ modules. By contrast, following the success of ViT, a novel research direction has emerged, designed from the Transformer backbone, to incorporate explicit convolutions or other desirable convolutional properties. For example, a layer-by-layer Tokens-To-Token (T2T) transformation^28^ was developed to gradually convert photos into tokens and produce local structural information. Further, they provided a T2T-ViT backbone with a deep-narrow architecture, which somewhat alleviated ViT’s reliance on large-scale datasets.^15^ proposed the Swin Transformer, which enables state-of-the-art methodologies in various CV tasks, such as image classification, object identification, and semantic segmentation, in addition to employing Transformers for image classification. Based on the Swin Transformer and to overcome the intrinsic locality limitations of convolutional operations, recently,^29^ proposed SwinE-Net, which effectively improved the robustness and accuracy of polyp segmentation by combining EfficientNet and Swin Transformer to maintain global semantics without sacrificing the low-level features of CNN.

Some researchers have proposed hybrid approaches that combine convolutional and SA modules in the same architecture instead of utilizing pure attention models. For example, the Convolutional Enhanced Image Transformer (CeiT)^30^ was introduced, which uses CNN to extract low-level characteristics before using the Transformer to construct long-range dependencies.^31^’s BoTNet combines the SA module into ResNet, allowing it to outperform ResNet in image classification and object identification tasks. Similarly,^18^ presented CoAtNet, a basic yet effective network structure made up primarily of MBConv blocks^32^ and Transformer blocks. Contrary to BoTNet, CoAtNet uses the MBConv block as the major component rather than the residual block, and the Transformer block is located in the last two stages rather than the final stage. CoAtNet can accomplish good generalization like CNN and superior model capacity like Transformer by employing this design. In addition,^33^ introduced the CNNs Meet Transformers (CMT) block, and^34^ proposed the convolutional ViT (CvT) architecture, which integrates convolutional layers with Transformers into a single block. The CMT and CvT designs, like ResNet^5^, contain multiple stages for generating feature maps of various sizes, each of which is made up of CMT/CvT blocks.

## Results

This section presents the experimental results obtained on the ODIR-5K dataset, comparing the proposed MBSaNet against diferent baselines.

### Implementation details

All experiments were performed on a dedicated server, the CPU is Intel Xeon Gold 6226R, 16 cores and 32 threads, the GPU is NVIDIA RTX5000, the memory is 32gb, and the GPU memory is 16 gb. To verify the effectiveness of the proposed model, we designed multiple sets of comparative experiments. We use the data-augmented original dataset for training, an off-site test set of 1,000 images, an on-site test set of 2,000 images, and a balanced test set of 400 images for testing. The hyperparameter settings are shown in Table 1.

**Table 1:** Hyperparameter settings.

Configuration	Value
Optimizer	Adam
Max epoch	30
BatchSize	32
Learning rate	1.00E-03,decay=1.00E-06
Batch normalization	True
Activation function	ReLu
Drop out	5.00E-01
EarlyStopping	Monitor=val$\_$loss,patience=5
ModelCheckpoint	Monitor=final$\_$score,mode=Max,
	restore$\_$best$\_$weights=True

**Table 2:** Comparison with some CNN networks and hybrid models on the off-site test set.

Model	Params	Accuracy	AUC	Kappa	F1$\_$Score	Final$\_$score
Vgg16^4^	134M	0.877	0.803	0.331	0.877	0.671
Vgg19^4^	139M	0.865	0.812	0.347	0.879	0.679
Inception-v3^35^	23.9M	0.878	0.873	0.323	0.877	0.691
ResNet50^5^	25.6M	0.875	0.836	0.387	0.875	0.699
MobileNetV2^32^	6.9M	0.882	0.781	0.302	0.869	0.651
Xception^36^	33M	**0.890**	0.860	0.344	0.874	0.693
EfficientNetB0^7^	5.9M	0.862	0.870	0.369	0.862	0.701
DenseNet^37^	27M	0.874	0.832	0.386	0.866	0.695
CoAtNet-0^18^	23M	0.869	0.689	0.102	0.862	0.551
CoAtNet-1^18^	40M	0.864	0.739	0.115	0.864	0.573
BotNet50^31^	20M	0.873	0.742	0.132	0.866	0.580
CoaT-Tiny^38^	7.7M	0.885	0.818	0.288	0.851	0.652
CoaT-Mini^38^	14.8M	0.872	0.806	0.266	0.853	0.642
MBSaNet (Ours)	9.4M	0.881	**0.891**	**0.438**	**0.881**	**0.737**

Significant values are in bold.

### Comparison experiment with CNNs and other hybrid models

Owing to the robust feature learning ability of CNNs, which avoids the tedious steps of manually designing features in traditional methods, CNNs have been the main model architecture for CV since the great breakthrough of AlexNet^39^. Recently some proposed CNN architectures have enabled models to attain state-of-the-art performance in tasks such as image classification and object detection in recent years. For performance testing, We compared MBSaNet with mainstream CNN backbone models on three independent test sets. The results showed that in the off-site test set, MBSaNet can achieve an AUC value of 0.891, a Kappa value of 0.438, an F1-score of 0.881, and a final score of 0.737; the CNN with the best performance in each indicator can achieve an AUC value of 0.870, a Kappa value of 0.369, an F1-score of 0.862, and a final score of 0.701. In the on-site test set, MBSaNet achieved an AUC value of 0.878, a Kappa value of 0.411, an F1-score of 0.884, and a final score of 0.724; meanwhile, the best performing CNN achieved an AUC value of 0.861, a Kappa value of 0.353, an F1-score of 0.863, and a final score of 0.692.

**Figure 1:**
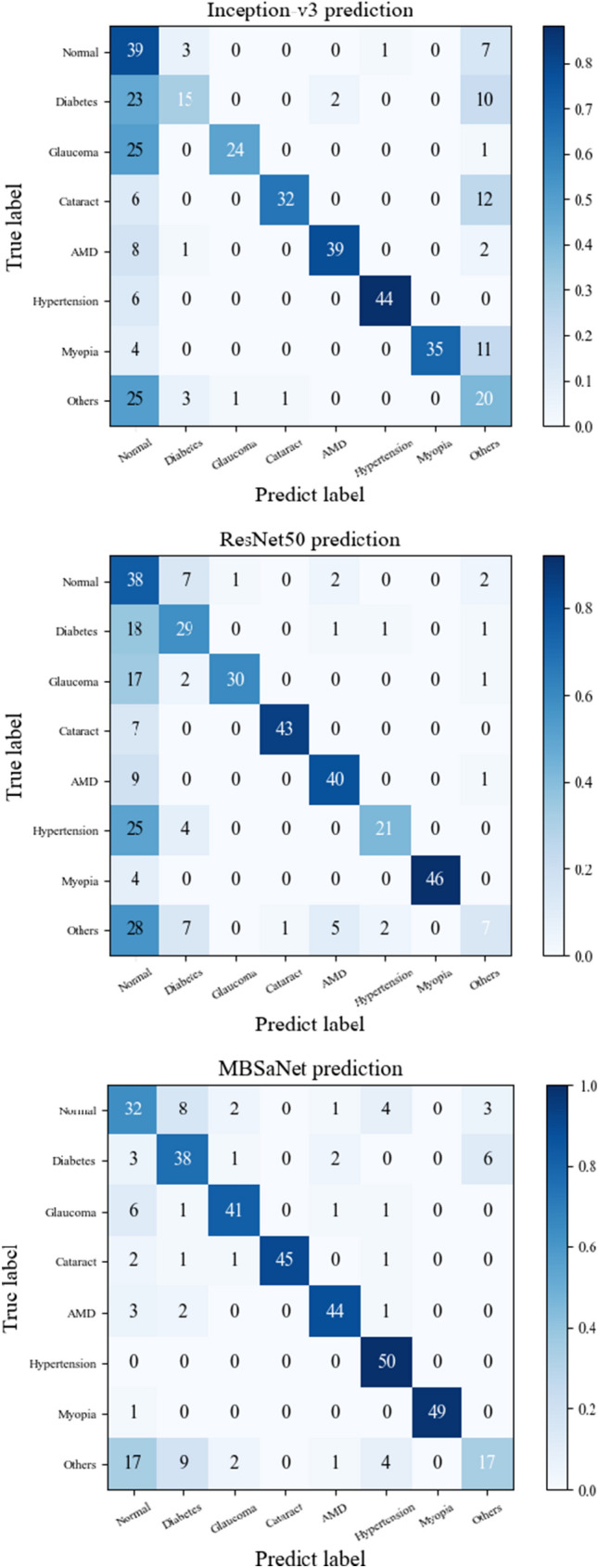
Classification results on the balanced test set.

On the balanced test set containing 400 images, MBSaNet achieved a precision of 0.50 and a recall of 0.64 for normal fundus, 0.64 and 0.76 for DR, 0.87 and 0.82 for glaucoma, 1.0 and 0.90 for cataract, 0.89 and 0.88 for AMD, 0.82 and 1.0 for hypertension, and 1.0 and 0.98 for myopia. The classification results of MBSaNet and the two best performing CNNs are shown in Figure 1.

Hybrid models based on CNN and Transformer have achieved state-of-the-art performance on large-scale datasets such as ImageNet, but they have not yet been applied in the field of fundus disease recognition with low image data quantity. To evaluate their performance and compare with MBSaNet, we conduct experiments with two Coat^38^ models, two different configuration models in the CoAtNet family^18^, and BotNet50^31^. To ensure fairness, we apply the parameter settings in Table 1 to all models and use the same data-augmented training set. The experimental results are shown in Tables 2, 3 and  4.

**Table 3:** Comparison with some CNN networks and hybrid models on the on-site test set.

Model	Params	Accuracy	AUC	Kappa	F1$\_$Score	Final$\_$score
Vgg16^4^	134M	0.874	0.799	0.334	0.859	0.664
Vgg19^4^	139M	0.872	0.791	0.328	0.865	0.661
Inception-v3^35^	23.9M	0.877	0.870	0.318	0.866	0.684
ResNet50^5^	25.6M	0.883	0.829	0.369	0.868	0.688
MobileNetV2^32^	6.9M	0.882	0.789	0.296	0.864	0.649
Xception^36^	33M	**0.887**	0.852	0.334	0.865	0.683
EfficientNetB0^7^	5.9M	0.859	0.861	0.353	0.863	0.692
DenseNet^37^	27M	0.865	0.842	0.350	0.862	0.684
CoAtNet-0^18^	23M	0.862	0.674	0.158	0.859	0.563
CoAtNet-1^18^	40M	0.857	0.701	0.166	0.861	0.576
BotNet50^31^	20M	0.863	0.734	0.138	0.863	0.578
CoaT-Tiny^38^	7.7M	0.879	0.810	0.284	0.833	0.642
CoaT-Mini^38^	14.8M	0.868	0.801	0.269	0.837	0.635
MBSaNet (Ours)	9.4M	0.879	**0.878**	**0.411**	**0.884**	**0.724**

Significant values are in bold.

**Table 4:** Comparison with some CNN networks and hybrid models on the balanced test set.

Model	Normal	DR	Glaucoma	Cataract	AMD	Hypertension	Myopia
**Vgg16**^4^							
Precision	0.31	0.31	0.83	0.78	0.56	0.55	0.89
Recall	0.30	0.34	0.40	0.94	0.76	0.82	0.96
**Vgg19**^4^							
Precision	0.24	0.32	0.84	0.88	0.72	0.49	0.89
Recall	0.32	0.36	0.52	0.94	0.52	0.88	0.94
**Inception-v3**^35^							
Precision	0.28	**0.68**	0.96	0.97	**0.95**	**0.97**	1.0
Recall	**0.78**	0.28	0.48	0.64	0.78	0.88	0.7
**ResNet50**^5^							
Precision	0.26	0.59	**0.97**	0.98	0.85	0.88	1.0
Recall	0.76	0.58	0.60	0.86	0.80	0.42	0.92
**Xception**^36^							
Precision	0.29	**0.68**	0.90	0.98	0.88	0.89	0.98
Recall	0.74	0.34	0.52	0.67	0.75	0.84	0.80
**DenseNet**^37^							
Precision	0.43	0.34	0.80	**1.0**	0.78	0.69	0.94
Recall	0.58	0.52	0.66	0.82	0.82	0.88	0.90
**CoaT-Tiny**^38^							
Precision	0.22	0.27	0.85	0.86	0.87	0.79	1.0
Recall	0.26	0.38	0.34	**0.96**	0.56	0.92	0.96
**MBSaNet (Ours)**							
Precision	**0.50**	0.64	0.87	**1.0**	0.89	0.82	**1.0**
Recall	0.64	**0.76**	**0.82**	0.90	**0.88**	**1.0**	**0.98**

Significant values are in bold.

### Comparison with previous work

In this subsection, the advanced nature of MBSaNet is verified by comparing it with several previous studies. Among them,^16^ proposed a model integration strategy, inputting the color and gray versions of the same fundus image into two EfficientNets with the same architecture for training, and integrating the output results of the two models to obtain the final output.^40^ used the Inception-v3^35^ model, replacing the network’s randomly generated weight parameters at the start of training with weight parameters that had been previously trained on ImageNet, and used the data-augmented image dataset for training. The experimental results on the off-site test set containing 1,000 fundus images are shown in Table 5.

**Table 5:** Comparison with previous works.

Study	Model	AUC	Kappa	F1$\_$score	Final$\_$score
jordi et al.^40^	Inception-v3	0.84	0.44	0.85	0.716
Wang et al.^16^	EfficientNetB3	0.73	0.50	0.88	0.703
Wang et al.^16^	EfficientNetB3	0.74	0.52	0.89	0.717
Gour and Khanna^41^	Two I/P VGG16	0.85	N/A	0.86	N/A
Our method	MBSaNet	0.89	0.44	0.88	0.737

### Ablation study

In this section, we investigate the effects of using various stacking schemes in the stem stage, and the performance impact of using global SA in the final stage of our multistage feature extraction network. The same settings as in Table 1 are used for a fair comparison.

**Figure 2:**
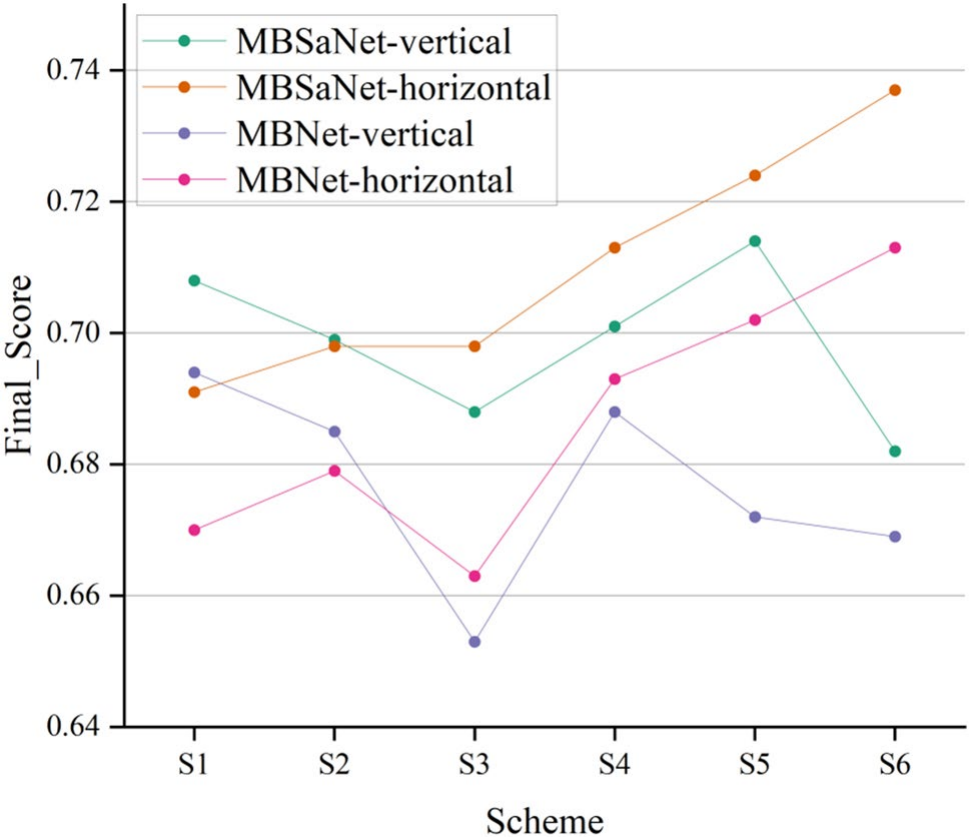
Performance of MBSaNet and its variant MBNet when using different convolution stacking strategies in the feature extraction stem stage.

We compare the performance of six different schemes of vertically stacked convolutions and horizontally stacked convolutions on the off-site test set. The specific combinations of the schemes are described in Table 6. The experimental results are shown in Figure 2, we demonstrating that stacking convolutions horizontally to widen the stem structure is more efficient than stacking convolutions vertically. Meanwhile, we observe the drop in metrics from replacing multiscale feature fusion stem (MFFS) with a single-scale feature fusion stem, and that using convolution kernels of different scales is more conducive to extracting high-quality features. By introducing an MBSaNet -variant, MBNet a network that uses only improved MBConv blocks, we verify the effectiveness of the global SA module. Based on the feature maps extracted in the convolution stage, a two-layer SA module is utilized in the final stage to further capture long-term dependencies, which significantly improves the feature modeling ability.

**Table 6:** Various schemes in the proposed MFFS for different stacking strategy.

Stacking strategy	S1	S2	S3	S4	S5	S6
Vertically	3,3	5,5	7,7	3,5	3,5,7	3,5,7,9
Horizontally	3,3	5,5	7,7	3,5	3,5,7	3,5,7,9

**Figure 3:**
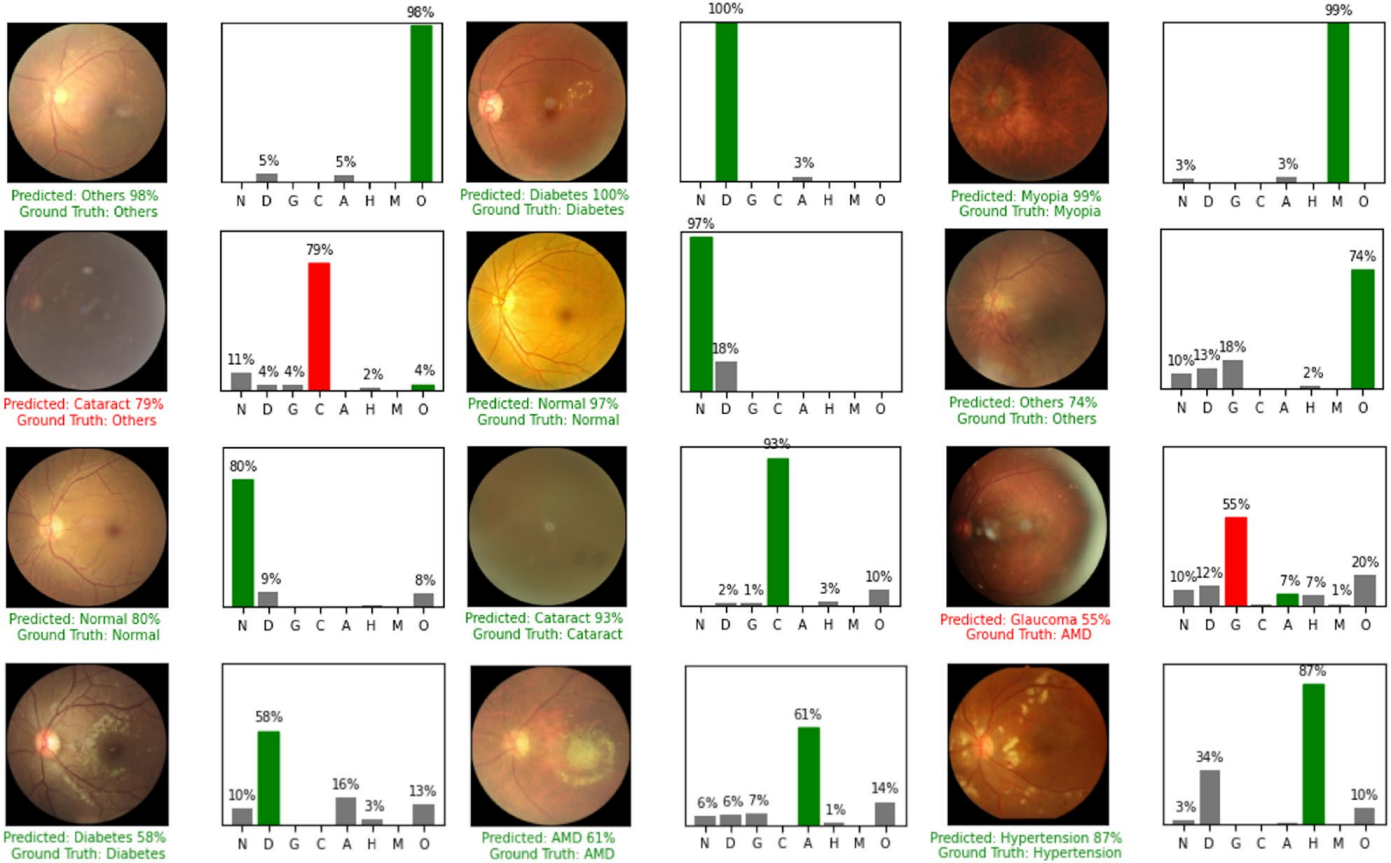
Some images evaluated by our model.

## Discussion

We introduced MBSaNet, a novel model based on the SA mechanism for fundus image classification, which is the first application of Transformer architecture in the field of fundus multidisease recognition, and hence provides a new idea for the research of SA models in the field of medical image processing. The experimental results showed that compared with many popular backbone networks, MBSaNet has higher accuracy in the recognition task of multiple fundus diseases. The wide range of image sources and the huge intra-category discriminations brought about by different camera acquisitions demonstrate the robust feature extraction capabilities of MBSaNet, indicating its great potential in assisting ophthalmologists in clinical diagnosis, especially in the identification of glaucoma, cataract, AMD, hypertensive retinopathy and myopic retinopathy. Figure 3 shows MBSaNet prediction results on some sample images from the test set.

By explicitly combining convolutional layers and SA layers in a multistage network, the model achieves a good balance between generalization performance and global feature modeling ability; while generalizing well on smaller datasets, high-quality semantic features can also be extracted from fundus images for decision-making by fully connected layers. From the experimental results, we can see that compared with the convolutional networks, MBSaNet achieved better performance with fewer parameters, in which the Kappa value was 5 percentage points higher than the best performing CNN model, indicating that MBSaNet’s prediction results are more consistent with the actual classification results, and the model is less biased toward categories , which makes sense on imbalanced datasets. In contrast, the accuracy metric is less relevant because there is a huge imbalance in the sample size of each category, and the model can obtain high accuracy by directly classifying the test sample into a category with large sample size.

We also compared MBSaNet with other hybrid models, and MBSaNet shows obvious advantages over other models. The poor performance of the other hybrid models on the fundus dataset can mainly be attributed to the fact that their generalization performance is not sufficient for the ODIR-5K dataset, although we have employed data augmentation techniques. Among them, although MBSaNet has a certain similarity with the CoAtNet models, there is a huge gap in the final score. We believe that this is mainly related to the use of SA modules in the last two stages of feature extraction in CoAtNet, no matter which configuration of CoAtNet, the stacking number of modules in the penultimate stage is the largest, and the amount of calculation is also the largest, choosing to use the SA module that lacks inductive bias, which will reduce the generalization performance of the model on smaller datasets. In addition, the number of hidden dimensions at each stage also affects the performance. In the experimental comparison with previous studies, on the three important metrics, AUC, Kappa, and F1-score, our MBSaNet only has a lower Kappa value than the model of^16^. Notably, the AUC value of MBSaNet far exceeds those of other models, considering that ODIR is an unbalanced dataset, and AUC is not sensitive to whether the sample size is balanced, it indicates that MBSaNet is a more ideal model for classification of multiple fundus diseases.

According to the prediction results of several models for balanced test set, the recognition accuracy for images with label O is generally poor, mainly because the label contains too many images of different categories, resulting in too large intra-class gap, making it difficult for the model to effectively partition them.

In ablation experiments, the networks with horizontally widened stems have better performance, and the network with MFFS achieves the best performance, which shows that this simple structure is effective, extracting image features at different scales and fusing them at the initial stage can help improve the classification performance. In addition, compared with the variant-MBNet, MBSaNet has better performance on all classification indicators, which indicates that by introducing the global receptive field and enhancing the global modeling ability of the model, the pathological features of different lesions in the fundus image can be extracted more effectively.

Due to the use of different camera equipment under different environmental conditions, the fundus images used in this study have high diversity. Hence, we adopted certain image preprocessing methods to Enhance contrast of the images features and expand the training dataset, on the premise of preserving the original image features as much as possible. Both raw and processed images are fed into the model for training, which can provide useful features for the identification of multiple fundus diseases. Some limitations of this study are as follows: (1) limited number of images in some categories may affect the performance of the model, although high diversity fundus images are used. (2) The distributions of categories in the on-site and off-site test datasets are unbalanced, and it is difficult to assess the classification accuracy of the model for a specific disease. (3) We eliminated a few images that were marked as low image quality, however, these images are unavoidable in practical situations. (4) It was found out that the effect of increasing the number of fully-connected layers of a neural networks depends on the type of data set being used^42^, in our experiments, we found that in the convolution stage, the number of hidden dimensions also has a great impact on the recognition accuracy of fundus diseases, which is worth further study.

## Methods

**Figure 4:**
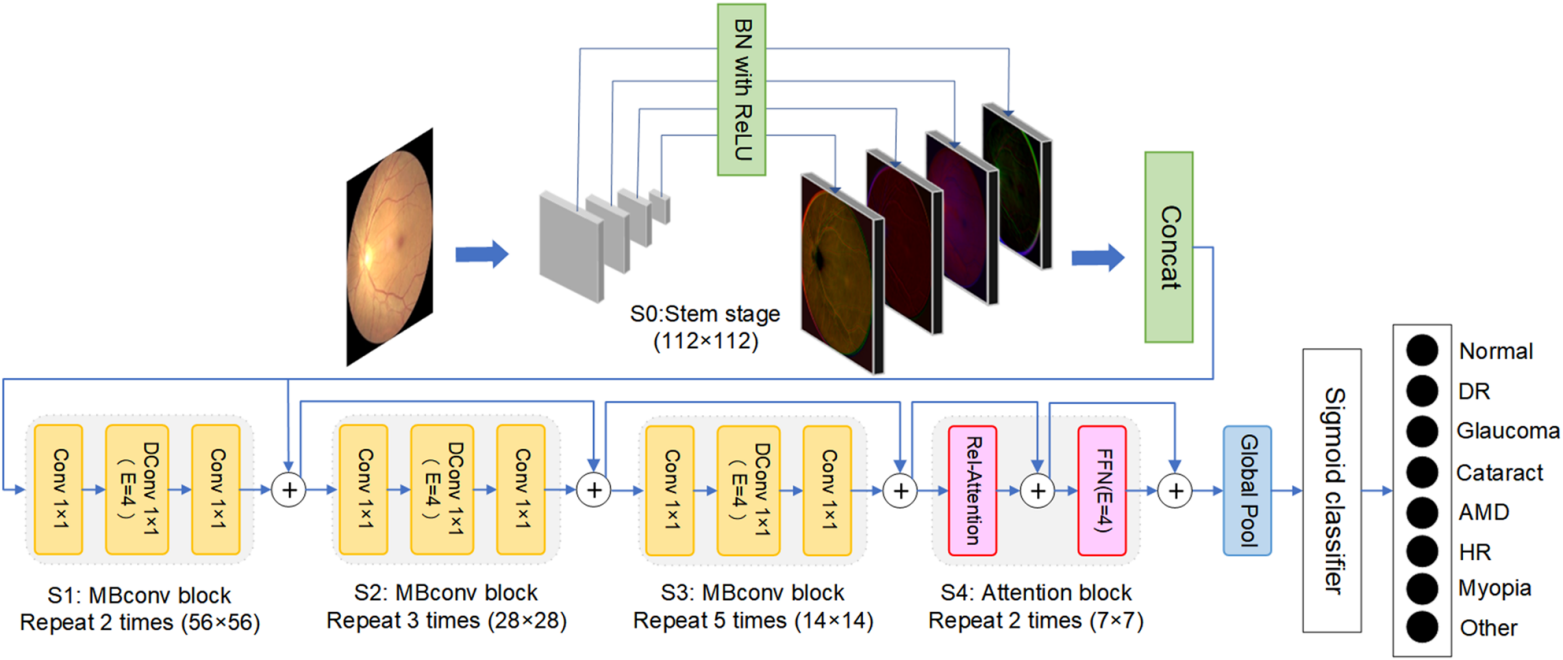
The overall architecture of MBSaNet.

MBSaNet is proposed to improve the performance of classification models on the task of automatic recognition of multilabel fundus diseases. The main idea of MBSaNet is based on the explicit combination of convolutional layers and SA layers, which enables the model to have both the generalization ability of CNN and the global feature modeling ability of Transformer^18,43^. Previous studies have demonstrated that the local prior of the convolutional layer makes it good for extracting local features from fundus images; however, we believe that long-term dependences and the global receptive field are also essential for fundus disease identification, because even an experienced ophthalmologist is unable to make an accurate diagnosis from a small part of a fundus image (e.g., using only a macula). Considering that the SA layer with global modeling ability can capture long-term dependencies, MBSaNet is implemented by adopting a building strategy similar to the CoAtNet^18^ architecture with vertically stacked convolutional blocks and self-attention modules. The overall framework of MBSaNet is shown in Figure 4, and Table 7 shows the size of the input and output feature maps at each stage of the model. The framework comprises two parts. The first of which is a feature extractor with five stages: Stage0–Stage4, where Stage0 is our proposed multiscale feature fusion stem (MFFS), Stage1–Stage3 are all convolutional layers, and Stage 4 is an SA layer with relative position representations. The second part is a multilabel classifier that predicts the sample category based on the features extracted from the above structure. We use the MBConv block that includes residual connections and an SE block^27^ as basic building blocks in all convolutional stages due to the same reverse bottleneck design as the Feedforward Network (FFN) block of Transformers. Unlike the regular MBConv block, MBSaNet replaces the max-pooling layers in the shortcut branch with convolutional layers having stride 2 in the downsampling strategy. This is a custom neural network that needs to be implemented by training it from scratch.

**Table 7:** The input and output feature map size of each stage.

Stage	Type	Input size	Output size
Stage0	Conv stem	224*224*3	112*112*64
Stage1	Conv block	112*112*64	56*56*128
Stage2	Conv block	56*56*128	28*28*256
Stage3	Conv block	28*28*256	14*14*512
Stage4	Attention block	14*14*512	7*7*512
Pooling	Global pool	7*7*512	1*1*512
Classifier	Full connection	512	8

**Figure 5:**
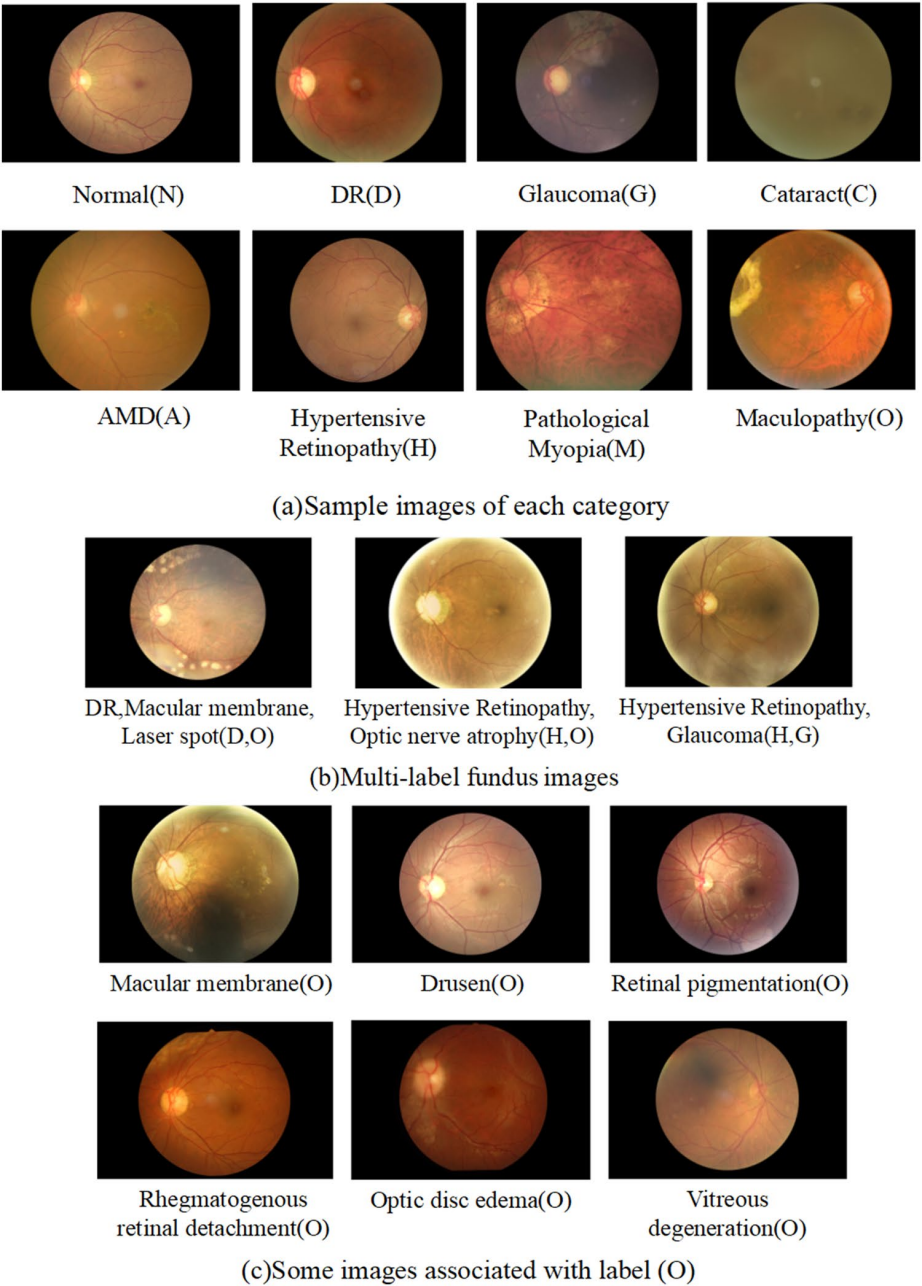
Sample images.

**Table 8:** Dataset summary.

dataset	No. of images	No. of individuals	Age Mean	Female no./total individuals($\%$)	Normal (left/right)	DR (left/right)	Glaucoma (left/right)	Cataract (left/right)	AMD (left/right)	H (left/right)	M (left/right)	O
Dataset for training	7,000	3,500	57.8	1615(0.461)	1533/1467	887/912	177/149	159/154	136/144	96/97	126/142	821
Off-site testing	1,000	500	58.2	231(0.462)	224/206	123/133	24/21	23/24	22/22	15/15	18/21	109
On-site testing	2,000	1,000	57.8	463(0.463)	416/403	241/245	42/41	45/50	37/40	25/29	33/35	318
Balanced test set	400				50	50	50	50	50	50	50	50

### Dataset

The dataset obtained from the “International Competition on Ocular Disease Intelligent Recognition” sponsored by Peking University. This dataset contains “real” patient data collected from different hospitals and medical centers in China, which were jointly launched by the Nankai University School of Computer Science-Beijing Shanggong Medical Information Technology Co., Ltd. joint laboratory. The training set is a structured ophthalmology database that includes the ages of 3,500 patients, color fundus images of their left and right eyes, and diagnostic keywords from clinicians. The test set includes off-site test set and on-site test set, but as with the training set, the number of samples under each category is unbalanced. Therefore, we also constructed a balanced test set with 50 images per class by randomly sampling a total of 400 images from the training set. The specific details of the dataset can be found in Table 8. Fundus images were recorded by various cameras, including Canon, Zeiss, and Kowa, with variable image resolutions. As illustrated in Figure 5(a), these data categorize patients into eight categories: normal (N), DR (D), glaucoma (G), cataract (C), AMD (A), hypertension (H), Myopia (M), and other diseases/abnormalities (O). There are two points to note. First, a patient may contain one or more labels, as shown in Figure 5(b), that is, the task is a multidisease multilabel image classification task. Second, as shown in Figure 5(c), the class labeled Other Diseases/Abnormalities (O) contains images related to more than 10 different diseases, and low quality images due to factors such as lens blemishes, and invisible optic discs, variability is largely expanded in. All the methods developed and experiments were carried out in accordance with the relevant guidelines and regulations associated to this publicly available dataset.

### Evaluation metrics

Accuracy is the proportion of correctly classified samples to the total samples, which is the most basic evaluation indicator in classification problems. Precision refers to the probability that the true label of a sample is positive among all samples predicted to be positive. Recall refers to the probability of being predicted by the model to be a positive sample among all the samples with positive labels, and given the specificity of the task, we use a micro-average of precision and recall for each category in our experiments. AUC is the area under the ROC curve, and the closer the value is to 1, the better the classification performance of the model. AUC is often used to measure model stability. The Kappa coefficient is another index calculated based on the confusion matrix, which is used to measure the classification accuracy of the model and can also be used for consistency testing, where p0 denotes the sum of the diagonal elements divided by the sum of the entire matrix elements, i.e., accuracy. pe denotes the sum of the products of the actual and predicted numbers corresponding to all categories, divided by the square of the total number of samples. F1$\_$score, also known as BalancedScore, is the harmonic (weighted) average of precision and recall, and given the category imbalance in the dataset, we use micro-averaging to calculate metrics globally by counting the total true positives,false negatives and false positives. The closer the value is to 1, the better the classification performance of the model. Final$\_$score is the average of F1$\_$score, Kappa, and AUC.

1 $$\begin{aligned} Accuracy= & {} \frac{TP+TN}{TP+FP+TN+FN} \end{aligned}$$

2 $$\begin{aligned} Precision= & {} \frac{TP}{TP+FP} \end{aligned}$$

3 $$\begin{aligned} Recall= & {} \frac{TP}{TP+FN} \end{aligned}$$

4 $$\begin{aligned} F1\_score= & {} \frac{2Precision*Recall}{Precision+Recall} \end{aligned}$$

5 $$\begin{aligned} Kappa= & {} \frac{p_0 - p_e}{1 - p_e} \end{aligned}$$

6 $$\begin{aligned} Final\_score= & {} \frac{F1\_score + Kappa + AUC}{3} \end{aligned}$$

### Data preprocessing

**Figure 6:**
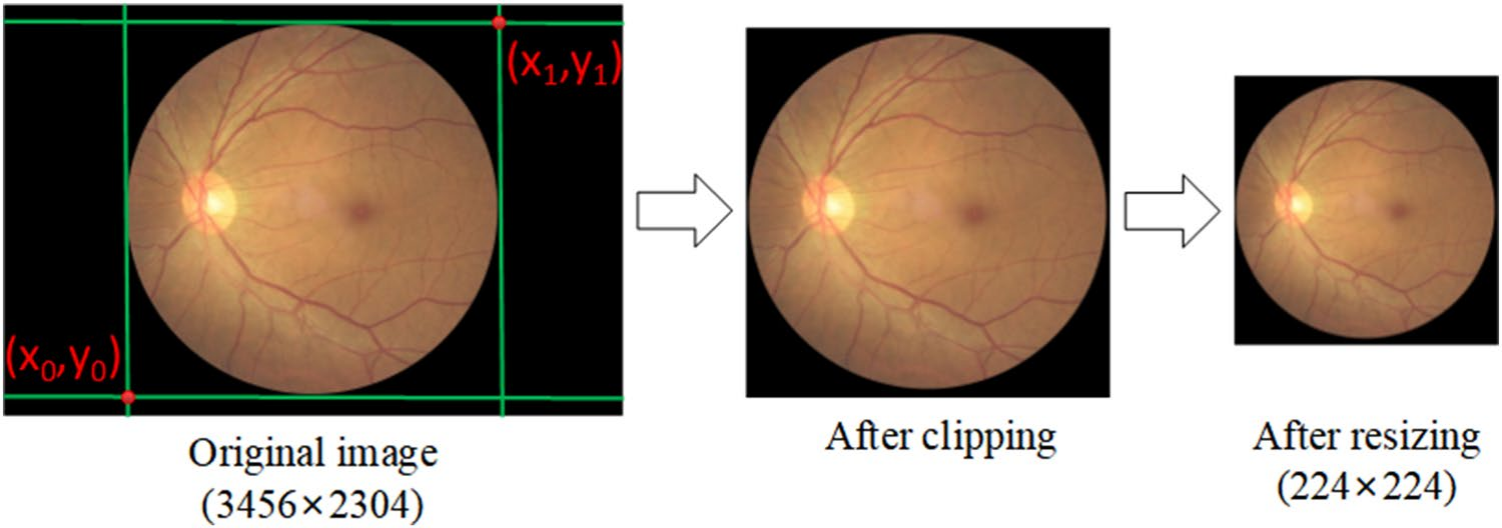
Processing of original training image.

**Figure 7:**
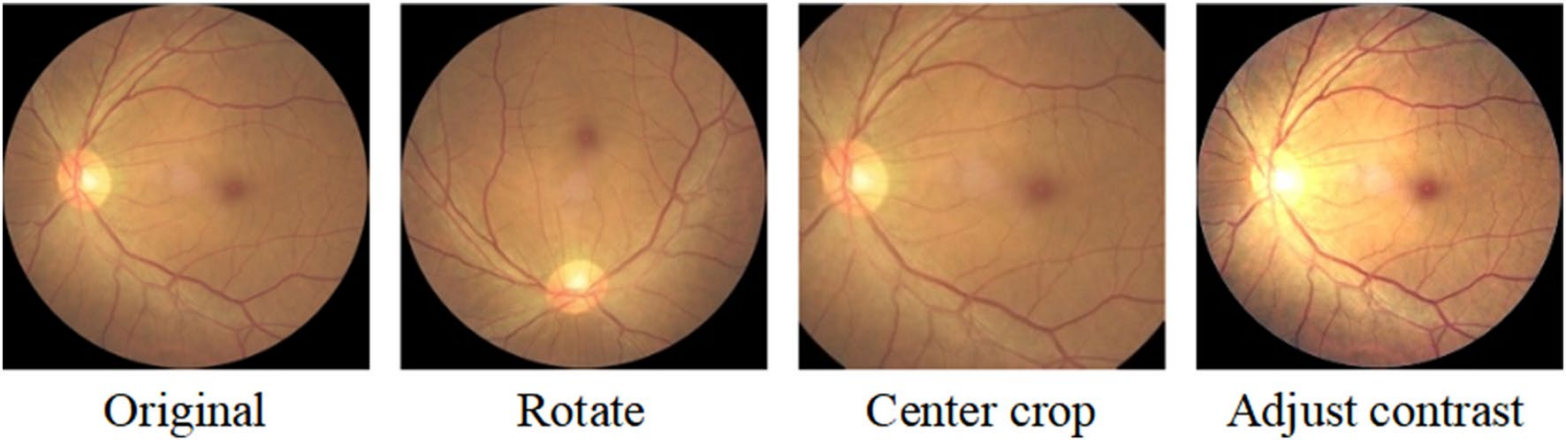
Data augmentation.

The fundus image dataset contains some low-quality images, which are removed since it would not be helpful for training. In order to minimize the unnecessary interference to the feature extraction process due to the extra noise brought by the black area of the fundus images, the redundant black area is cropped. We use the OpenCV library to load the image as a pixel vector and use the edge position coordinates of the retinal region of the fundus image to remove the black edges. The fundus images are further resized to a 224×224 image size after being cropped as shown in Figure 6. Data augmentation is the artificial generation of different versions of a real dataset to increase its data size; the images after data augmentation are shown in Figure 7. Because it is necessary to expand the size of the dataset based on retaining the main features of the original image, we use operations such as random rotation by 90$^\circ$, adjustment of contrast, and center cropping. Finally, the global histogram equalization operation is performed on the original and enhanced images, so that the contrast of the images is higher and the gray value distribution is more uniform.

### Multiscale feature fusion stem

The predictive ability of a classifier is closely related to its ability to extract high-quality features. In the field of fundus multidisease identification, owing to the different characteristics of the lesions reflected in the fundus images of several common eye diseases, the lesion areas have the characteristics of different sizes and distributions. We propose a feature fusion module with convolution kernels of different sizes to extract multiscale primary features of images in the input stage of the network and fuse them in the channel dimension. Feature extractors with convolution kernel sizes of 3$\times$3, 5$\times$5, 7$\times$7, and 9$\times$9 are used, since the convolution stride is set to 2, we padding the input image before performing each convolution operation to ensure that the output feature maps are the same size. By employing convolution kernels with different receptive fields in the horizontal direction to broaden the stem structure, more locally or globally biased features are extracted from the original images. The batch normalization operation and ReLU activation are then performed separately and the resulting feature maps are concatenated. The experimental results show that by widening the stem structure in the horizontal direction, higher quality low-level image features can be obtained at the primary stage.

### Multistage feature extractor

**Figure 8:**
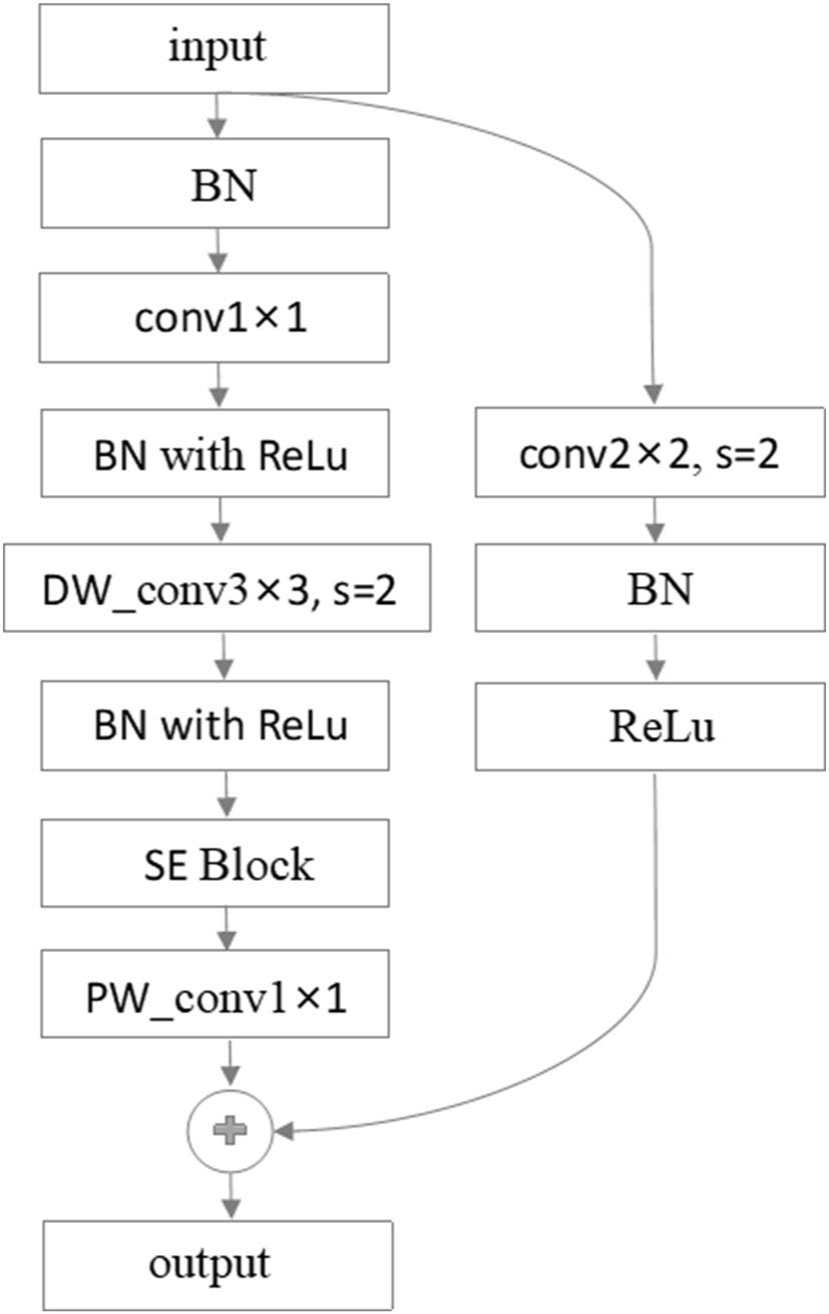
Convolutional building blocks.

CNNs have been the dominant structure for many CV tasks. Traditionally, regular convolutional blocks, such as ResNet blocks^5^, are well-known in large-scale convolutional networks; meanwhile, depthwise convolutions^44^ can be expressed as Formula 7 and are popular on mobile platforms due to their lower computation cost and smaller parameter size. Recent studies have shown that an improved inverse residual bottleneck block (MBConv)^32,45^ which is built on depthwise separable convolutions can achieve both high accuracy and efficiency^7^. Inspired by the CoAtNet^18^ framework, we consider the connection between the MBConv block and FFN module in the Transformer (both adopt the inverted bottleneck design: first expand the feature map to 4$\times$ the size of the input channel, and after the depth separable convolutions operation, project the 4$\times$ wide feature map back to the original channel size to satisfy the residual connection), and mainly adopt the improved MBConv block including the residual connection and SE^27^ block as the convolution building block. The convolution operation with a convolution kernel size of 2$\times$2 and a stride of 2, implements the output feature map size on the shortcut branch to match the output size of the residual branch. The experimental results show that this slightly improves the performance. The convolutional building blocks we use are shown in Figure 8, and the downsampling implementation can be expressed as Formula 8.

7 $$\begin{aligned} y_i = \sum _{j\in {\mathcal {L}} (i)}^{} w_{i-j} \odot x_j \quad \quad {(\mathrm depthwise\quad \mathrm convolution)} \end{aligned}$$

where $x_i,y_i \in {R}^{D}$ denote the input and output at position *i*, respectively, and ${\mathcal {L}} (i)$ denotes a local neighborhood of *i*, e.g., a 3$\times$3 grid centered at *i* in image processing.

8 $$\begin{aligned} \mathrm {x\longleftarrow Norm(Conv(x,stride=2))+Conv(DepthConv(Conv(Norm(x),stride=2)))} \end{aligned}$$

In natural language processing and speech understanding, the Transformer design, which includes a crucial component of the SA module, has been widely used. SA extends the receptive field to all spatial places and computes weights based on the re-normalized pairwise similarity between the pair $(x_i,x_j)$, as shown in Formula 9, where ${\mathcal {G}}$ indicates the global spatial space. Stand-alone SA networks^33^ have shown that diverse CV tasks may be performed satisfactorily using SA modules alone, albeit with some practical limitations, in early research. After pretraining on the large-scale JFT dataset, ViT^11^ applied the vanilla Transformer to ImageNet classification and produced outstanding results. However, with insufficient training data, ViT still trails well behind SOTA CNNs. This is mainly because typical Transformer architectures lack the translation equivalence^18^ of CNNs, which increases the generalization on small datasets^46^. Therefore, we decided to adopt a method similar to CoAtNet; the global static convolution kernel is summed with the adaptive attention matrix before softmax normalization, which can be expressed as Formula 10, where (*i*, *j*) denotes any position pair and $w_{i-j}$ denotes the corresponding convolution weights, improve the generalization ability of the network based on the Transformer architecture by introducing the inductive bias of the CNNs.

9 $$\begin{aligned} y_{i}= & {} \sum _{j \in {\mathcal {G}}} \underbrace{\frac{\exp \left( x_{i}^{\top } x_{j}\right) }{\sum _{k \in {\mathcal {G}}} \exp \left( x_{i}^{\top } x_{k}\right) }}_{A_{i, j}} x_{j} \end{aligned}$$

10 $$\begin{aligned} y_{i}^{\text{ pre } }= & {} \sum _{j \in {\mathcal {G}}} \frac{\exp \left( x_{i}^{\top } x_{j}+w_{i-j}\right) }{\sum _{k \in {\mathcal {G}}} \exp \left( x_{i}^{\top } x_{k}+w_{i-k}\right) } x_{j} \end{aligned}$$

The receptive field size is one of the most critical differences between SA and convolutional modules. In general, a larger receptive field provides more contextual information, but this usually results in higher model capacity. The global receptive field has been a key motivation for employing SA mechanisms in vision. However, a larger receptive field requires more computation. For global attention, the complexity is quadratic *w*.*r*.*t*. spatial size. Therefore, in the process of designing the feature extraction backbone, considering the huge computational overhead brought by the Transformer structure and the small amount of training data for practical tasks, we use more convolution blocks, and only set up two layers of SA modules in Stage4 in the feature extraction stage. Experimental results show that this achieves a good balance between generalization performance and feature modeling ability.

### Multilabel loss function

The fundus disease recognition task is a multilabel classification problem, so it is unsuitable for training models with traditional loss functions. We refer to the loss function used in work^16,40^, all classified images can be represented as $$X = $${$$x_1,x_2...x_i...x_N$$} , where $$x_i$$ is related to the ground truth label $$y_i$$, and $$i = 1...N$$, *N* represents the number of samples. We wish to find a classification function $$F:X\longrightarrow Y$$ that minimizes the loss function *L*, we use N sets of labeled training data $$(x_i,y_i)$$, and apply a one-hot method to each $$y_i$$ is encoded, $$y_i = [y_i^1,y_i^2...y_i^8] $$, each *y* contains 8 values, corresponding to the 8 categories in the dataset. We draw on the traditional multilabel classification method based on problem transformation, and transformed the multilabel classification problem into a two-class classification problem for each label. The final loss is the average of the loss values of the samples corresponding to each label. After studying weighted loss functions, such as sample balance and class balance, we decided to use weighted binary cross-entropy from Formula 11 as the loss function, where *W* = (1,1.2,1.5,1.5,1.5,1.5,1.5,1.2) denotes the loss weight. The positive class is 1, and the negative class is 0. $$p(y_i)$$ is the probability that sample *i* is predicted to be positive.

11 $$\begin{aligned} L=-\frac{1}{N} \sum _{i=1}^{N} W \left(y_{i} \log \left( p\left( y_{i}\right) \right) +\left( 1-y_{i}\right) \log \left( 1-p\left( y_{i}\right) \right) \right) \end{aligned}$$

After obtaining the loss function, we need to choose an appropriate optimization function to optimize the learning parameters. Different optimizers have different effects on parameter training, so we mainly consider the effects of SGD and Adam on model performance. We performed multiple comparison experiments under the same conditions. The results showed that Adam significantly outperformed SGD in terms of convergence and shortened training time, possibly because when we chose SGD as the optimizer, the gradients of the samples were updated at every epoch, which brings additional noise. Each iteration is not in the direction of the global optimum, so it can only converge to the local optimum, decreasing accuracy.

## Data Availability

The datasets used to train our models and run experiments is available, upon registration from the ODIR-2019 Challenge https://odir2019.grand-challenge.org/. And for further research in this area we have made the code available at https://github.com/ironchelsea/MBSaNet.
